# A case of eagle fern (*Pteridium aquilinum*) poisoning on a pig farm

**DOI:** 10.1186/s40813-020-00185-9

**Published:** 2021-01-04

**Authors:** Agnès Waret-Szkuta, Laura Jégou, Marie Noelle Lucas, Nicolas Gaide, Hervé Morvan, Guy-Pierre Martineau

**Affiliations:** 1 IHAP, Université de Toulouse, INRA, ENVT, Toulouse, France; 2 LABOCEA, Service Bactériologie Vétérinaire, Ploufragan, France

**Keywords:** Eagle fern, Mortality, Outdoor rearing, Vitamin B1, Intoxication

## Abstract

**Background:**

Free-range pig farming represents a minor proportion of pig production in France but is attracting an increasing number of farmers because of societal expectations and the opportunity to use pasture-grazed forage. However, this type of farming faces several challenges, including biosecurity, parasitic management, and contact with wild fauna and pathogenic flora.

**Case presentation:**

Two Gascon pigs raised on an outdoor fattening farm in the Hautes-Pyrenees department of France were submitted after sudden death for necropsy at the National Veterinary School of Toulouse. The pigs were of two different breeds but from the same group of 85 animals that had grazed on a 4-ha plot of land being used for grazing for the first time. Based on an in-depth interview with the farmer, the epidemiological information available, and the necropsy and histology examinations, a hypothesis of great eagle fern intoxication was proposed. Although the sample of animals available for diagnosis was small, the success of the administered therapy confirmed our diagnosis. It was recommended that in the short term, the animals be prevented access to the eagle fern by changing their pasture or removing the plants. Vitamin B1 and vitamin B6 were administered via feed as Ultra B® at 1 mL per 10 kg body weight per day for 2 days (providing 9 mg thiamine (vitamin B1) and 0.66 mg pyridoxine (vitamin B6) per kg body weight per day). Marked remission was observed, with 6 of 10 intoxicated animals with symptoms surviving (yielding a therapeutic success rate over 50%), but the therapy did not compensate for the loss of initial body condition. In total, of the 85 animals in the group after intoxication, 6 died, and 6 recovered.

**Conclusions:**

The significance of this report lies in the scarcity of eagle fern intoxication cases reported in the literature, though such intoxication may become a significant problem as the development of outdoor rearing continues. Thus, eagle fern intoxication should be included in the differential diagnosis of nervous system symptoms in swine. The case also emphasizes the importance of anamnesis and discussion with the farmer as an essential step to guide diagnosis.

## Background

Today, free-range pig farming represents 5% of pig production in France [1]. There are several challenges, including climate, which may impact feed conversion [2]; contact with the soil, which may promote parasitic infection; potential interactions with wild animals; and potential ingestion of toxic plants [3]. As this type of farming is attracting an increasing number of farmers because of societal expectations [4] and the opportunity to use pasture-grazed forage [5, 6], it is likely that cases of plant poisoning following ingestion of plants such as eagle fern will increase in frequency. However, reports of such cases to date have been rare.

Great eagle fern (*Pteridium aquilinum*), also called bracken fern, is an invasive plant that grows globally in very diverse environments and is difficult control in pastures [7]. Intoxication following ingestion is well documented in ruminants, including cattle, sheep and wild cervids [8, 9]. In cattle, bracken fern ingestion causes enzootic haematuria, characterized by lower urinary tract haemorrhages and neoplasms of epithelial and mesenchymal origin [9]. Additionally, acute haemorrhagic syndrome secondary to bone marrow aplasia and retinal atrophy have been documented in sheep [8], as has polioencephalomalacia [10].

Bracken fern poisoning is poorly documented in monogastric species. In horses, poisoning has been associated with the ingestion of poor-quality hay containing a high proportion of big eagle fern [11], the use of the fern as bedding [12] or overgrazing [13]. Case reports in pigs are rare [14–16], probably because outdoor rearing of pigs is less prevalent than that of other livestock; they might also be rare due to the high resistance of pigs, which can ingest this plant without being exposed to the poison for a period of 6 weeks [10].

Bracken fern contains several toxins, including ptaquiloside, thiaminase, quercetin and bleeding factors [17, 18]. These toxic molecules, ptaquiloside and thiaminase in particular, are found in high concentrations in the young growing parts of the plant and in the rhizomes. Thiaminase is an enzyme that breaks down thiamine (the essential vitamin B1) into pyrimidine and thiazole. Since vitamin B1 is essential for metabolism and for maintenance of peripheral nerve myelin [10], vitamin B1 deficiency leads to peripheral or central neuropathy, with manifestations including impaired health status, weight loss and slight loss of movement coordination [11]. Symptoms suggestive of heart failure and anorexia have also been reported. Cardiopathy related to vitamin B1 deficiency has been studied in pigs to understand the pathogenesis of beriberi, a human disease caused by a nutritional deficiency in vitamin B1. One study showed that thiamine deficiency was associated with nonhypertrophic cardiac dilatation and histopathological lesions, including focal to diffuse necrosis of the myocardium and neutrophilic and mononuclear myocarditis [19].

The purpose of this report is to describe a case of great eagle fern poisoning with sudden deaths in one of eight groups on an outdoor Gascon pig-fattening farm in the Hautes-Pyrenees department of France. The affected group of 85 pigs had been reared on a new pasture of 4 ha.

## Case presentation

The case described here occurred on an outdoor fattening farm with 680 Gascon pigs in the Hautes-Pyrenees department of France. Every 2 or 3 months, a group of 85 animals weighing approximately 30–40 kg arrive from a breeding farm and remain on the same plot until they are approximately one-year-old, with an average carcass weight of 140 kg. The plot is then subjected to a 2-month period without pigs on it. Pigs from eight groups are present simultaneously. The animals are fed triticale flour and consume forage they encounter in the environment: acorns, chestnuts, grass, brambles, heather and elements from soil excavation. Water is provided as well water, which does not undergo any chemical treatment. The animals are vaccinated against *Erysipelas rhusiopathiae* intra-muscularly upon arrival and receive a booster injection 1 month later. To prevent parasites, the farmer adds Panacur 4%® to the drinking water at a dosage of 12.5 g per 100 kg of body weight every 2 months. Biosecurity is limited. (There is no electric fencing, but wires are present.)

In October 2019, the farmer noticed losses of body condition and appetite in approximately ten pigs from a single plot. Breathing difficulties with an open mouth and complaints were observed in many animals, as well as nervous system signs that manifested as a wobbling gait. There was no diarrhoea. Two pigs that died suddenly were brought for necropsy to the Veterinary School of Toulouse. One pig had been found dead 3 weeks prior. These animals came from the same group of 85 animals and had been grazing on a 4-ha plot of land being grazed for the first time.

The clinical signs reported by the farmer were partially non-specific, with signs of weight loss and loss of appetite, but this suggests a less acute evolution than the sudden mortality reported concomitantly. The nervous system symptoms suggested central nervous damage with a wide variety of potential aetiologies, both infectious (e.g., oedema disease) and non-infectious (nutritional deficiency or intoxication). The respiratory symptoms similarly may have had various causes and may or may not have been related to the nervous system symptoms. Parasites can also lead to death and are particularly important to consider in cases of an outdoor setting; such parasites include *Metastrongylus* spp. (e.g., *Metastrongylus apri*) and *Trichuris* spp. (e.g., *Trichuris suis*). However, since the plot on which the pigs were located was being used for the first time for fattening and since parasite treatment was administered regularly, this hypothesis was not retained at first.

Epidemiologically, the phenomenon appeared that it could be contagious or anazootic. Since the cases were limited to a single group of pigs at the time of the call, with 15% morbidity (13/85), 3.5% mortality (3/85) and 23% lethality (3/13), a contagious origin was not retained as first. However, given the occurrence of African swine fever in Europe, particular attention was paid not to overlook the possible detection of this disease, especially as biosecurity was not optimal at the outdoor farm and as the wild boar population in the region is increasing [20].

### Post-mortem investigation

**Fig. 1:**
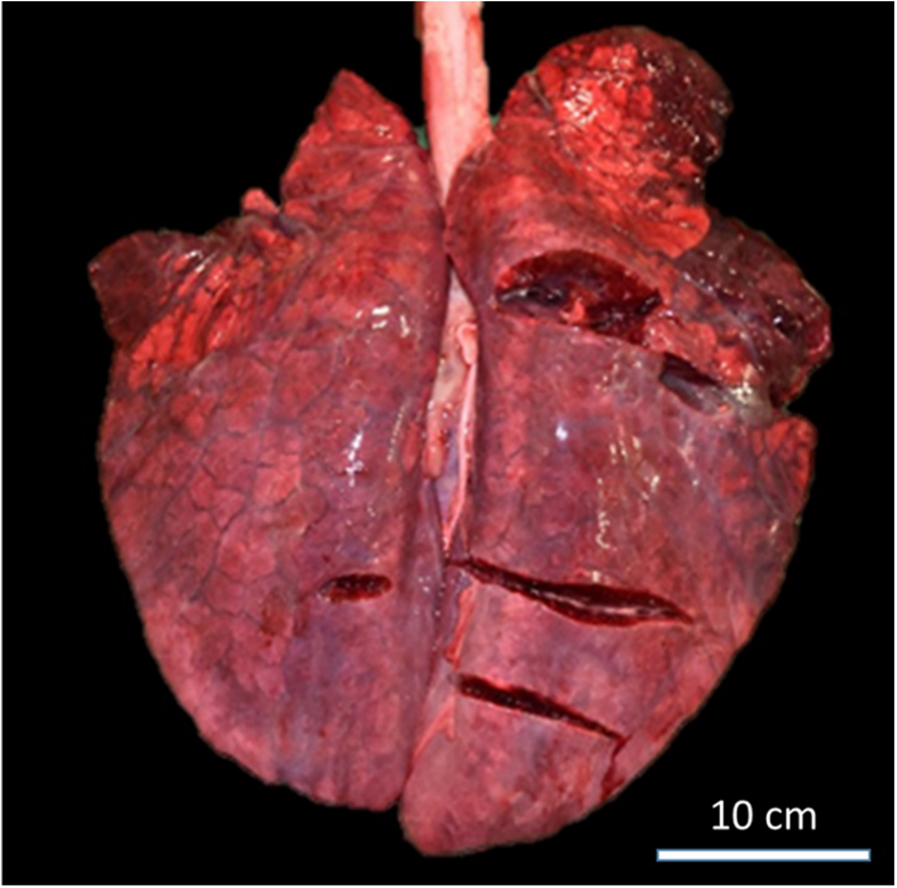
Lung with moderate cranio-ventral consolidation and marked diffuse interlobular oedema

Two pigs that were found dead were submitted for complete necropsy: an 8-month-old, 89-kg sow and an 8-month-old, 59-kg male pig that died the day before. External examination of the sow revealed congestive ocular and oral mucosa. Cavity openings revealed 500 mL and 15 mL yellow translucent effusion consistent with transudate in the peritoneal and pericardial cavities, respectively. The lung presented a moderate cranio-ventral consolidation and marked diffuse interlobular oedema (Fig. 1).

**Fig. 2:**
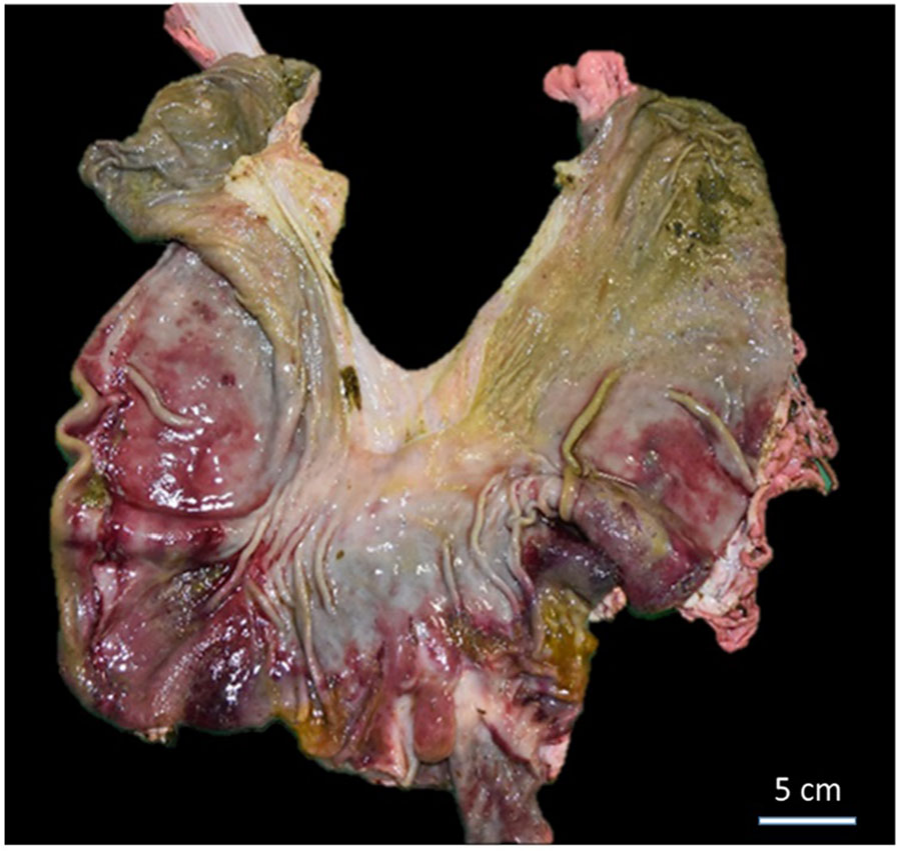
Stomach with extensive fundic congestion

The mediastinal lymph nodes were enlarged, measuring 2.5 × 1 cm and 3.5 × 1 cm. Extensive fundic congestion (Fig. 2) as well as foamy liquid in the tracheal lumen and diffuse petechiae on the diaphragm was found.

On the male pig, gross lesions were limited to 70 mL of yellow pericardial effusion, diffuse fundic congestion and multifocal foci of congestion in the caecal mucosa without luminal abnormalities. The rest of the examined organs were within normal limits, including the gross external examination of the brain and meninges.

The post-mortem examinations did not reveal any specific lesions supporting a definitive diagnosis of an infectious agent except in the lung, where the lesions suggested potential enzootic bronchopneumonia (*Mycoplasma hyopneumoniae*), and an underlying acute diffuse inflammatory lesion that did not allow the ruling out of a parasitic or viral interstitial pneumonia. At the time of necropsy, the brain, liver, and heart of both animals and the lung, kidney, mediastinal and mesenteric lymph nodes of the sow were harvested and fixed in 10% buffered formalin. After fixation, they were routinely embedded in paraffin blocks, sectioned into 4-μm slices and stained with haematoxylin and eosin (HE) for microscopic evaluation.

At the same time, the farmer was contacted again to gather more information on the animals’ pasture environment, in particular, on the possible neighbourhood issues and the plants in the plot, considering that the plot was being used for the first time for pig fattening, the presence of nervous system signs, and the absence of clinical signs in the other pig groups present on the farm, all of which were on plots that had been used previously for pig farming.

**Fig. 3:**
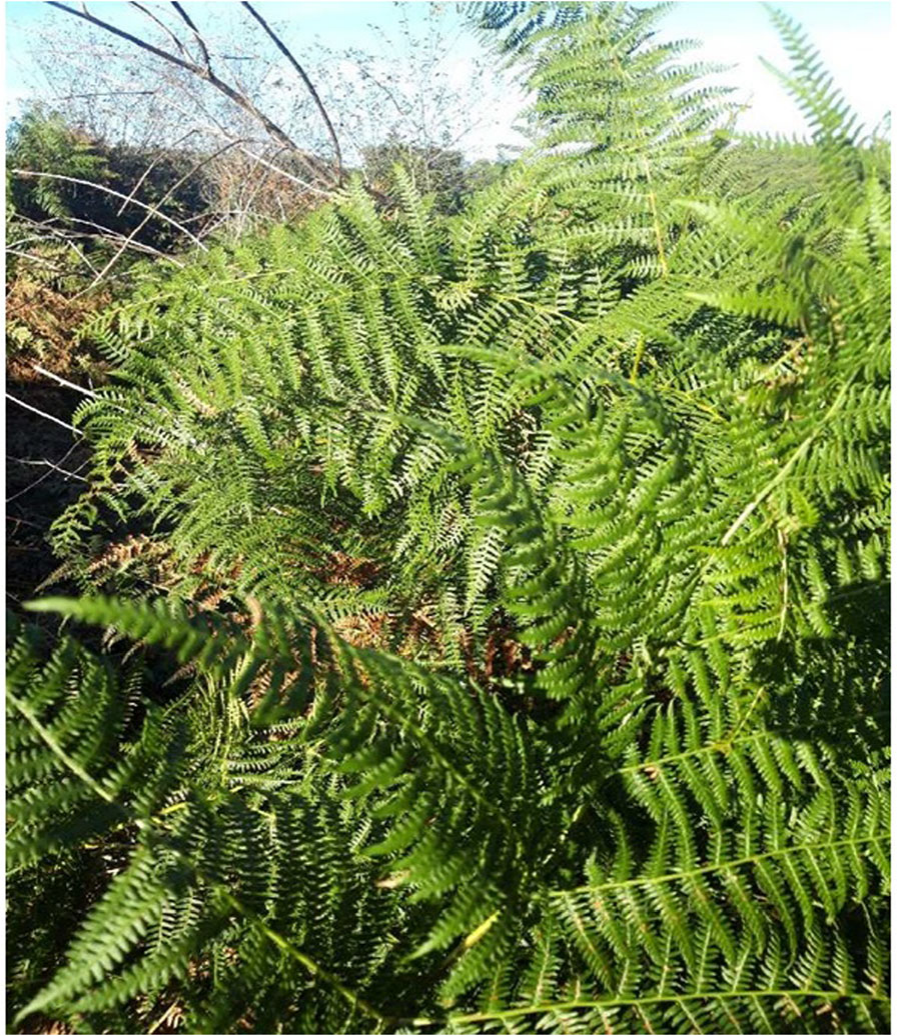
Great eagle fern (*Pteridium aquilinum*)

This second in-depth interview with the farmer revealed the abundant presence of great eagle fern (*Pteridium aquilinum*) in the stockyard, which was confirmed by photographs sent by the farmer (Fig. 3).

At that point, the lack of specificity of the necropsy findings associated with the presence of the fern without any change in the animals’ management, particularly regarding the feed distributed, led to a strong suspicion of plant intoxication by this fern, which can lead to thiamine deficiency.

### Histology

**Fig. 4:**
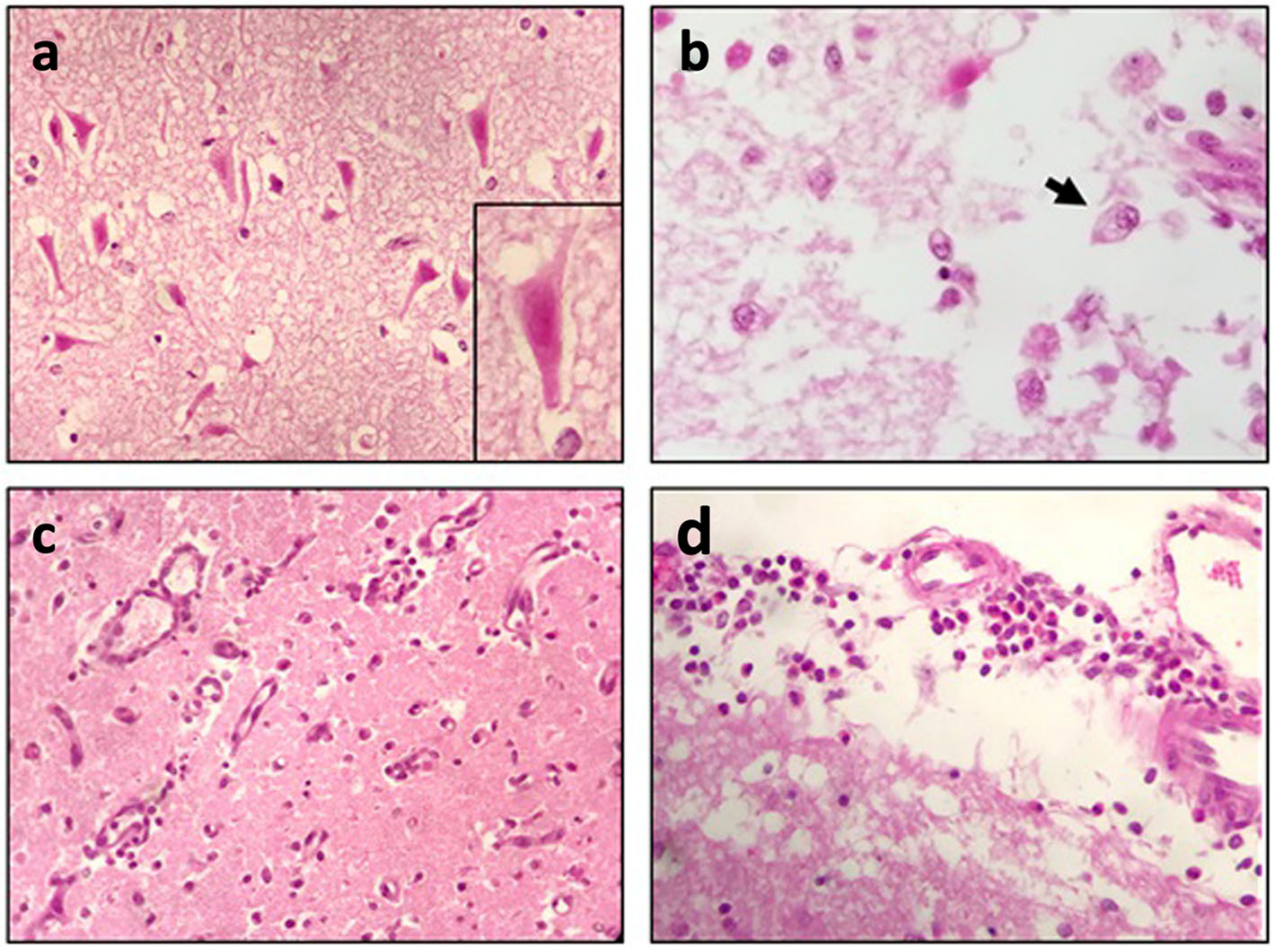
Histological photographs of brain of the male pig. **a** Cerebrocortical acidophilic neuronal necrosis (× 400). **b** Gitter cells (arrow) in the spongiotic area (× 400). **c** Endothelial activation with perivascular leukocytic infiltration (× 200). **d** Leptomeningeal leukocytic infiltration, including eosinophil infiltration (× 400)

Samples taken from the male pig showed no abnormalities except in the brain. Lesions consisted of segmental cortical laminar and multifocal lesions in the brainstem, basal nuclei, and cerebellum, which were associated with acidophilic neuronal necrosis and spongiosis and admixed with gitter cell infiltration (Fig. 4). Inflammation was observed in the leptomeninges and perivascular spaces, and endothelial activation, hyperaemia, and mixed leukocytic infiltration involving mononuclear cells and eosinophils were observed. Vascular fibrinoid necrosis and haemorrhages were occasionally seen. A diagnosis of polioencephalomalacia was assessed, suggesting a toxic or metabolic aetiology, including a toxic plant or a thiamine deficiency.

**Fig. 5:**
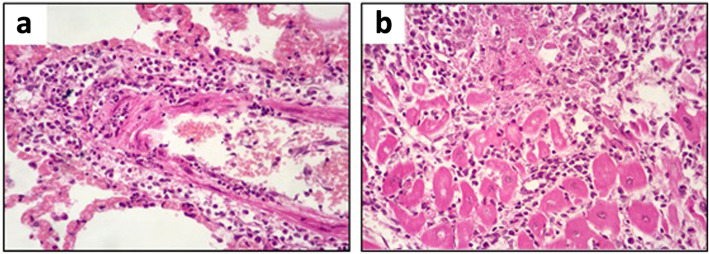
Histological photographs of lung and heart of the female pig. **a** Lung with necrotizing leukocytoclastic vasculitis. **b** Heart with polyphasic necrotizing and leukocytic myocarditis (HE, × 400)

In the sow, the brain, lymph nodes, liver and kidneys were within normal limits. The heart showed lesions consistent with severe progressive polyphasic necrotizing myocarditis with mixed leukocytic infiltration, and the lungs showed leukocytoclastic necrotizing vasculitis with diffuse congestive and oedematous pneumonia (Fig. 5).

A systemic infectious origin, notably viral, such as porcine circovirus type 2 (PCV2), was suggested based solely on histological findings without evidence of pathognomonic lesions, such as basophilic inclusions or granulomatous inflammation, and with myocardial lesions being found more often in runts or small piglets. Despite the non-specificity, intoxication could not be ruled out, since pulmonary vasculitis was previously described in bracken fern intoxication of pigs [16].

### Diagnostic and recommendations

Considering anamnesis, epidemiology, and post-mortem investigations and histology, the most likely diagnostic hypothesis retained was intoxication by great eagle fern. This hypothesis was confirmed by the good response to the therapy that was implemented.

It was recommended that in the short term, the animals be prevented access to the eagle fern by changing their pasture or by removing the plants. Vitamin B1 and vitamin B6 were administered in the feed as Ultra B® at 1 mL per 10 kg body weight per day (providing 9 mg thiamine (vitamin B1) and 0.66 mg pyridoxine (vitamin B6) per kg body weight per day) for 2 days according to the SPCs of the product. Oral administration was preferred to injection because of the outdoor setting and the potential exposure of the whole group to eagle fern. All of the animals maintained on the affected plot were treated. Pronounced remission was observed, with 6 of the 10 intoxicated animals with symptoms surviving following treatment administration (yielding a therapeutic success rate of over 50%), but the treatment did not compensate for the loss of initial body condition. Of the 85 animals in the group 6 animals died following intoxication (before or after treatment), and 6 recovered. The animal that had died 3 weeks before the farmer first reported the events on the farm was not considered to share the same aetiology.

## Discussion and conclusion

Here, we report a case of eagle fern intoxication in the rearing of post-weaning, free-range fattening Gascon pigs. The significance in this case lies in the scarcity of cases reported in the literature. This case emphasizes the importance of anamnesis and discussion with the farmer as an essential step to guide diagnosis.

Clinicopathological manifestations of bracken fern intoxication in pigs remain poorly described. Clinically, loss of appetite, respiratory distress, signs of backward heart failure, rapid deterioration and sudden death have been reported [7, 16, 21–23]. Only one report mentioned ataxia prior to death [23]. Predominant findings on post-mortem examination include pulmonary oedema without evident signs of consolidation or pleuritis, hydropericardium and diffuse myocardial pallor [16]. Harwood and collaborators (2007) reported histological lesions of heart and lung consistent with necrotizing severe subacute myocarditis as well as pulmonary congestion and oedema with focal fibrinoid vasculitis in one pig [16]. Altogether, the pathological description of the present report is consistent with the literature, with strong myocardial and pulmonary involvement, but also included polioencephalomalacia and non-suppurative meningitis. Nervous lesions of bracken fern poisoning in pigs are poorly documented in the literature, but cortical laminar necrosis and meningitis is consistent with thiamine deficiency, as previously reported in pigs and ruminants [24].

In cases of bracken fern poisoning, drug treatment with thiamine (vitamin B1) should be administered as early as possible, but its effect is often reported to be limited in all species [13]. In horses, the recommended dose is 500 mg to 1 g/day of thiamine intravenously on the first day and then intra-muscularly for several days [11]. However, in our case, the administration of 9 mg thiamine (vitamin B1) and 0.66 mg pyridoxine (vitamin B6) per kg body weight per day in the feed was followed by marked remission, yielding a therapeutic success rate of over 50%, although without compensation for the loss of initial body condition. Vitamin B6 was administered concomitantly to vitamin B1 as no market-authorized product for swine containing only vitamin B1 was available at short term. Oral administration was preferred to injections considering the outdoor setting and potential exposition of the whole group to eagle fern.

In the medium and long term, the aim is to reduce and remove great eagle fern. Its growth can be slowed by regular mowing several times a year, although this technique is time consuming [25]. A fern-crushing roller is more effective, and its light weight allows emerging broadleaf and grassy plants to grow and compete with the fern. In addition, there is little damage to wildlife as a result of using this tool. The fern-crushing roller remains the best tool on the market to eradicate this fern and can be used on all soils. The best time for its use is between June and July, when the maximum rhizome reserves have been mobilized [26]. This method is effective but time consuming, with the effects of rolling only becoming visible after 2 or 3 years of double rolling (end of June and end of July) [13]. A study conducted in 2006 and 2007 tested an alternative treatment based on 12% vinegar, which achieved a good response in 2006 but was followed by regrowth in 2007 only a few months after treatment [27]. The authors of that study hypothesized that the soil moisture content during application differed between the 2 years. In the same study, a hexazinone treatment was utilized and yielded good results. However, this product is a broad-spectrum herbicide and is therefore not applicable in our context, since the animals graze in the pasture throughout the year [28]. Therefore, for the time being, the mechanical method is the most suitable method for the control of great eagle fern.

Although the therapeutic success supported our main hypothesis as to the most likely cause of the clinical signs in this case, some laboratory analyses, such as stomach content analysis, and the examination of a greater number of animals post-mortem could have strengthened our diagnosis before the implementation of a therapy. Indeed, our diagnosis was based on the necropsy of two animals out of 85, i.e., 2% of the group, and one can doubt the representativeness of the lesions observed in the individuals examined. Luckily, the African swine fever hypothesis could be ruled out despite enlarged mediastinal lymph nodes and diffuse petechiae on the diaphragm on one of the two pigs necropsied, which could have been lesions caused by the virus. Indeed, no other pigs became diseased, and the treated animals recovered. However, considering the present epidemiological situation of African swine fever in Europe, mortalities on an outdoor farm that has non-optimal biosecurity against interactions with wild boars should be thoroughly investigated via laboratory testing and other approaches. Such investigation should be conducted considering the possible route of transmission of the virus and that the clinical course and associated lesions of the disease may vary. Passive surveillance via farmers and veterinarians is of major importance for early detection in domestic and wild pigs to control spread of the virus [29, 30]. Furthermore, in the present case, considering the clinical history of sudden death, the lung oedema or fluids in the body cavities and the histopathological findings of neuronal necrosis, perivascular and meningeal inflammation and severe myocarditis, the hypothesis of encephalomyocarditis could have been investigated. However, the pattern of cerebrocortical necrosis did not suggest neutropic viruses, which produce a multifocal asymmetrical distribution of inflammation, and no clinical case of encephalomyocarditis confirmed by immunohistochemistry on heart sections has been reported in France to date. The observed infiltration of eosinophils, vascular necrosis and polioencephalomalacia could suggest salt intoxication or water deprivation, but severe myocarditis and vasculitis were not present, and water was being provided as well water. Porcine circovirus type 2 (PCV2) was also considered in relation to the histological findings but was not included in the initial list of possible diagnoses and thus was not specifically considered when collecting samples during the necropsies. However, oedema disease is considered as part of the differential diagnosis of sudden death with neurological disorders. This disease is caused by *Escherichia coli* strains that produce the Shiga toxin (STx2e) and express F18 fimbriae. In our case, neurological clinical signs and the histopathological findings of spongiosis and vascular fibrinoid necrosis were in favour of this hypothesis, whereas the age of the animals and the absence of macroscopic systemic oedema were not. As part of a potential complementary investigation to investigate this possibility, we could have attempted to isolate the F18 antigen and STx2e toxin from the intestinal contents. Furthermore, although septicaemia could have been suspected because of the clinical signs and petechial bleeding in the diaphragm, the number of animals affected made this diagnosis unlikely. In addition, possible nutritional deficiencies could have been discussed with the farmer, as the growth of the pigs could probably have been improved and vitamin B1 deficiencies can occur by malnutrition, although such cases remain uncommon.

Although few cases of pig eagle fern intoxication have been reported in the literature, this case demonstrates that this hypothesis should be included in the differential diagnosis of nervous system symptoms and/or sudden death in swine, particularly in the context of outdoor rearing. In view of the development of this type of farming in response to strong societal demand, it is highly likely that diseases of parasitic or toxicological types will become increasingly important in this species in the future. To investigate such diseases, site visits would be beneficial to visualize the facilities in place for the animals. Furthermore, as in our case, in-depth discussions with the farmer are crucial to establish a diagnosis.

## Data Availability

Data sharing is not applicable to this article, as no datasets were generated or analysed during the current study.

## Abbreviations

HE: Haematoxylin and eosin
PCV2: Porcine circovirus type 2

## References

[1] Delsart M, Pol F, Dufour B, Rose N, Fablet C. Pig farming in alternative systems: strengths and challenges in terms of animal welfare, biosecurity, animal health and pork safety. Agriculture. 2020. 10.3390/agriculture10070261.

[2] Lahrmann HK, Bremermann N, Kaufmann O, Dahms S (2004). Health, growing performance and meat quality of pigs in indoor and outdoor housing--a controlled field trial. Dtsch Tierarztl Wochenschr.

[3] Roepstorff A, Mejer H, Nejsum P, Thamsborg SM (2011). Helminth parasites in pigs: new challenges in pig production and current research highlights. Vet Parasitol.

[4] Roguet C, Neumeister D, Magdelaine P, Dockes AC (2017). Les débats de société Sur l’élevage au sein de l’Union européenne : thèmes, arguments et modes d’action des parties prenantes, conséquences Sur les modes d’élevage. Journées de la Recherche Porcine.

[5] Park HS, Min B, Oh SH (2017). Research trends in outdoor pig production — a review. Asian-Australas J Anim Sci.

[6] Roinsard A, Bordes A, Calvar C, Maupertuis F, Alibert L, Ferchaud S, Uzereau A, Roinsard A, Carriere J (2014). Valorisation des ressources fourragères par les porcins. Alimentation des porcins en agriculture biologique, eds ITAB, IBB, Chambre d'agriculture des pays de loire, IFIP.

[7] Vetter J (2009). A biological hazard of our age: bracken fern [Pteridium aquilinum (L.) Kuhn]--a review. Acta Vet Hung.

[8] Cortinovis C, Caloni F (2013). Epidemiology of intoxication of domestic animals by plants in Europe. Vet J Lond Engl.

[9] Scala C, Ortiz K, Catinaud J, Lemberger K (2014). Hematuria and urinary bladder lesions compatible with bracken fern (*Pteridium aquilinum*) intoxication in captive fallow deer (*Dama dama*). J Zoo Wildl Med Off Publ Am Assoc Zoo Vet.

[10] Stegelmeier BL (2014). Overview of bracken Fern poisoning - toxicology. Merck Veterinary Manual.

[11] Wright B. L’intoxication des chevaux par la fougère d’aigle. http://www.omafra.gov.on.ca/french/livestock/horses/facts/09-050.htm . Accessed 03 Jul 2020.

[12] Priymenko N. Végétox’ root. http://www.vegetox.envt.fr/. Accessed 02 Jul 2020.

[13] Gallet S (2002). Le contrôle de la fougère-Aigle.

[14] Banrie. Bracken poisoning in pigs, 2013. The pig site. https://thepigsite.com/articles/bracken-poisoning-in-pigs. Accessed 08 Sept 2020.

[15] Edwards BL. Poisoning by *Pteridium aquilinum* in pregnant sows. Vet Rec. 1983. 10.1136/vr.112.19.459. 10.1136/vr.112.19.459 6868319

[16] Harwood DG, Palmer NM, Wessels ME, Woodger NG. Suspected bracken poisoning in pigs. Vet Rec. 2007. 10.1136/vr.160.26.914-c. 10.1136/vr.160.26.914-c 17602110

[17] Evans A, Humphreys DJ, Goulden L, Thomas AJ, Evans WC (1963). Effects of bracken rhizomes on the pig. J Comp Pathol.

[18] Maxie G (2015). Jubb, Kennedy & Palmer’s pathology of domestic animals-E-book: volume 2 (Vol. 2). Elsevier health sciences.

[19] Follis RH, Miller MH, Wintrobe MM, Stein HJ (1943). Development of myocardial necrosis and absence of nerve degeneration in thiamine deficiency in pigs. Am J Pathol.

[20] Collongues JL. En Hautes-Pyrénées les sangliers ont occasionné 40 fois plus de dégâts aux cultures. *La Dépêche*, 21/08/2019. https://www.ladepeche.fr/2019/08/21/en-hautes-pyrenees-les-sangliers-ont-occasionne-40-fois-plus-de-degats-aux-cultures,8370150.php. Accessed 27 Jul 2020.

[21] Evans WC, Patel MC, Koohy Y (1982). Acute braken fern poisoning in homogastric and ruminant animals. Proc R Soc Edinb. Section B. Biol Sci.

[22] Fenwick GR (1989). Braken (Pteridium aquilinum) - toxic effect and toxic constituents. J Sci Food Agric.

[23] Defra pig expert group. Braken poisoning in pigs. http://apha.defra.gov.uk/documents/surveillance/diseases/bracken-poisoning-pigs.pdf. Accessed 19 Nov 2020.

[24] Hough SD, Jennings SH, Almond GW (2015). Thiamine-responsive neurological disorder of swine. J Swine Health Prod.

[25] Hannah T, Michaud H (2006). Contrôler le développement de la fougère Aigle. Espaces naturels.

[26] Jurado C, Martinez-avilez M, De la Torre A, Stukeli M, De Carvalho Ferreira HC, Cerioli M, Sanchez-Vizcaino JM, Bellini S (2018). Relevant measures to prevent the spread of African swine fever in the European Union domestic pig sector. Front Vet Sci.

[27] Gagnon S (2009). Essai et expérimentation Sur la pollinisation et la réduction des herbicides dans la production du bleuet Nain au Saguenay–lac-saint-Jean.

[28] Gama A (2006). Utilisation des herbicides en forêt et gestion durable.

[29] Dixon LK, Stahl K, Jori F, Vial L, Pfeiffer DU (2020). African swine fever epidemiology and control. Annu.Rev.Anim.Biosci..

[30] Salguero FJ. Comparative pathology and pathogenesis of African swine fever infection in swine. Front Vet Sci. 2020. 10.3389/fvets.2020.00282. 10.3389/fvets.2020.00282 PMC7248413 32509811

